# Examining dimensions of teachers’ digital competence: A systematic review pre- and during COVID-19

**DOI:** 10.1016/j.heliyon.2023.e16677

**Published:** 2023-05-26

**Authors:** Bjørn Smestad, Ove Edvard Hatlevik, Monica Johannesen, Leikny Øgrim

**Affiliations:** a OsloMet, Oslo Metropolitan University, Oslo, Norway; b Volda University College, Norway

**Keywords:** Teachers' digital competence, Conceptual understanding, Teacher role, Transdisciplinary competency, COVID-19

## Abstract

The digitisation of education has heightened the importance of examining which competences are needed among teachers and student teachers. In the past decade, the opportunities and challenges related to using digital technologies in teaching and training have made the concept of ‘digital competence’ increasingly relevant. This paper examines how researchers have characterised the dimensions of teachers' digital competences both before and during the COVID-19 pandemic. In a literature review, 116 articles were analysed to identify prevalent understandings of teachers' and student teachers' digital competence. The search was conducted in two rounds: the period up to and including 2019 and supplements from 2020 to 2021. The latter search focused on literature addressing school closures because of ‘lockdowns’. The findings indicate that research on teachers' digital competence seems unclear regarding who benefits from teachers' digital competence, the teacher's role and the links between competence and school subject domains. Moreover, teachers have a more functional role than a designer role. In addition, studies on digital competence are typically based on self-reported data, and most publications that have examined the concept of digital competence include knowledge, skills or attitudes. The COVID-19 pandemic seems to have increased the focus on the whole group of pupils and on the use of ready-made educational designs. The pandemic may also have increased researchers' reliance on self-reported data.

## Highlights

• The study identifies the characteristics of research on teachers' professional digital competence.
• The literature seems unclear about who benefits from teachers' digital competence.
• The literature also seems unclear about teachers' role and the role of school subjects.
• Current studies on digital competence are typically based on self-reported data.
• The lockdown seems to have increased the focus on ready-made educational designs.

## 1 Introduction

The digitisation of education can impact the development of teachers' digital competence and their perceptions of this area of competence [1]. The concept of digital teaching is transdisciplinary and is often described as consisting of pedagogical and methodological knowledge, content knowledge of school subjects and transdisciplinary knowledge [2,3]. Recent review articles have addressed the training of teachers' digital competence [4], the development of digital competence [5] and digital competence among university teachers [6]. However, deeper insights into how the concept of teachers' digital competence is presented in the literature is lacking. The present review looks more closely at the understanding of teachers' professional digital competence in education research. Smestad and Gillespie [7] identified the characteristics of teachers' competences in three transdisciplinary areas: (i) digital competence, (ii) competence in diversity and (iii) competence in research and development. Based on a systematic review of 380 articles, they identified six dimensions of teachers' professional competence in which there were tensions in the literature. The current paper builds on this review and further explores a particular transdisciplinary competence: teachers' professional digital competence. The original search for research papers was performed in 2019. Subsequently, we extended the review to include papers from 2020 to 2021 to determine whether school closures because of the COVID-19 pandemic influenced the understanding of the concept. Only papers that explicitly discussed home schooling and the teaching situation during such closures were included in this additional search. The dimensions described by Smestad and Gillespie [7] have been used as a framework to analyse various aspects of teachers' professional digital competence. Because the dimensions are identified in a review of studies on three different competences, they may provide novel lenses through which we can study teachers’ digital competence.

The two research questions are as follows.

**RQ1**: What characterises the dimensions identified in research on teachers' digital competence?
**RQ2:***To what extent does this characterisation also apply to the literature on teachers' digital competence in connection with the COVID-19 pandemic?*

## 2 The concept of digital competence

This section presents relevant research on and definitions of ‘digital competence’, which is a key concept in achieving digitalised schools and societies. Several different notions, such as digital competencies [3,8], digital skills [9] and digital literacy [[10], [11], [12]], have been used to describe approximately the same concept. Many attempts have been made to define the concept [[13], [14], [15]]. According to Smestad and Gillespie [7], a typical feature of the papers describing teachers' digital competence is the use of models to define, describe, categorise and measure such competence. Examples of such models are TPACK [2], DigCompEdu [3] and PEAT [16].

The present study seeks to offer novel insights into the diverse dimensions of the conceptualisations of teachers' digital competence in academic research. In the research on this topic, these conceptualisations can wield a significant influence, even when they are not explicitly deliberated. Thus, our aim is to contribute to the discourse surrounding this issue. Furthermore, there has been a proliferation of studies focusing on teachers' digital competence in light of the COVID-19 pandemic. Thus, our study examines how the conceptualisation of teachers' digital competence may have differed during (or been altered by) the pandemic. By utilising those dimensions developed based on three distinct transdisciplinary competences, our study reveals perspectives that may be obscured when viewed solely from within the field of teachers’ digital competence.

## 3 Dimensions of teachers’ digital competence

Teachers' digital competence has often been described and defined in terms of practical user skills that are deemed essential for such competence [see Refs. [3,5,17]]. Self-reported data have frequently been used to measure teachers' actual digital competence [18,19]. However, the current study aims to reveal prevalent understandings of teachers' digital competence within the literature rather than identifying its contents/elements or measuring teachers’ self-reported levels of competence.

In discussing teachers’ competences within three transdisciplinary areas, Smestad and Gillespie [7] referred to six dimensions in which they identified tensions in discussions on competences.

D1: The *beneficiary* dimension concerns what an article addresses regarding who benefits from teachers' having a competence.
D2: The *teachers' role* dimension concerns which teacher roles are emphasised in the articles reviewed.
D3: The *attitudes, knowledge and skills* dimension concerns how studies focus on different components of competences.
D4: The *sources of the competence* dimension are what competences are based on.
D5: The *relationship to the disciplinary content* dimension relates to disciplinary subjects as the core of a competence.
D6: The *assessment* dimension focuses on the process of identifying and assessing teachers' competences.

Smestad and Gillespie (2020) suggested looking closer at the content of these dimensions.

## 4 Methods

As mentioned, Smestad and Gillespie [7] investigated conceptualisations of teachers' competences in three areas: diversity, research and development and digital competence. The Smestad and Gillespie study was a systematic review, specifically a concept synthesis [see Ref. [20]]. One result was a list of ‘six key dimensions within which there were tensions in the literature reviewed’: beneficiary; teachers' role; attitudes, knowledge and skills; sources of competence; relationship to disciplinary content; and assessment [ [7], p. 125–6]. In the current article, we have built on the part of their review discussing teachers' digital competence and supplemented the articles with newer ones related to teaching during the COVID-19 pandemic. The original and new searches were almost identical, except for the period covered. However, the searches used different inclusion criteria.

### 4.1 Research design

The following steps can be used to describe the research design of the review [21].

Step 1: Research questions were formulated (see section 1). RQ1 addresses the dimensions identified in research on teachers' digital competence, and RQ2 addresses the literature on teachers' digital competence related to the COVID-19 pandemic.
Step 2: Search words and strings were developed, and relevant databases were identified (see section 4.2). To answer RQ1, the search approach from a previous study [7] was appropriate, and this approach was used. However, to answer RQ2, an additional search had to be carried out.
Step 3: Criteria for inclusion and exclusion were developed to examine the quality and relevance of the studies from the search (see section 4.3). The quality of the studies informs decisions regarding inclusion or exclusion in the review.
Step 4: Summarising the evidence by synthesising the data. An analytical framework was adopted (see section 3), and this framework was further operationalised through reading, analysing and categorising the publications (see sections 5 and 6).
Step 5: Interpreting the findings to answer the research questions (see sections 6 and 7.2). It is also important to discuss the limitations of the study (see section 7.1) and further need for research based on our findings (see section 7.3).

### 4.2 Search strategies

The search was originally performed for the study documented in Smestad and Gillespie [7] and was conducted in the international databases Education Resources Information Center (ERIC), Education Source, Teacher Reference Center and Web of Science. Four Scandinavian databases, that is, Swepub, the Danish National Research Database, Norart and Idunn, were also included. We included articles published in English, Norwegian, Danish and Swedish. The search had three main elements related to *teachers*, *competence* and *the digital realm*. The years 2014–2019 were included in the original search, while the additional search included studies from 2020 to June 2021. The additional search included ‘COVID-19’ and ‘pandemic’ as search strings. For full details of the original search strategies, see Smestad and Gillespie [ [7], pp. 121–122 and its appendix]. Because the Danish National Research Database was discontinued in January 2021, this database was excluded from the additional search.

### 4.3 Inclusion and exclusion criteria

We excluded articles that did not explicitly signal in the title or abstract that they discussed teachers’ competence. Moreover, we excluded articles wherein the teachers were not mainly classroom teachers of grades 1–10 pupils (ages 6–16 years). Texts that were not articles, not written in English or a Scandinavian language were outside the defined time frames or discussed specific challenges of developing countries were also excluded. In the additional search, we included only articles mentioning the pandemic in the title or abstract.

In the original search, a team of six researchers used Rayyan software to review titles and abstracts. The researchers first reviewed the same 100 titles and abstracts. After fine-tuning the inclusion and exclusion criteria, the titles and abstracts of the remaining articles were read by only one researcher. In cases of doubt, the abstracts were read by two researchers. In the additional search, a team of four members reviewed the articles. Again, only one researcher read the title and abstract of each article, except when in doubt. In the readings of the full texts, only one reviewer read each text and consulted the others when in doubt.

### 4.4 Analysing and coding the articles based on the six dimensions

We analysed the selected articles based on a coding scheme developed in two phases. In this review of the teachers' digital competence, we lent ourselves to the dimensions proposed by Smestad and Gillespie [7]. We first developed an initial version of the categories of teachers’ digital competence based on the descriptions of the six dimensions in Smestad and Gillespie [7]. Then, the articles were divided among the researchers, and we discussed and developed the coding scheme after reading a few articles each. This process was repeated several times. The resulting categories function as analytic coding schemes.

The categories developed within the six dimensions are as follows.

D1: Within the *beneficiary* dimension, we identified categories demonstrating whether an article explicitly illustrates that an *individual* or *group of pupils* (e.g., a class) can benefit from teachers' digital competence. It could also be that *society* benefits from teachers' competence and that the competence includes their ability to connect to their pupils' *contexts* (e.g., home and family, culture or religion).
D2: The *teachers' role* dimension concerns which teacher role is examined in an article. For example, some studies view the teacher as a *functionary in* implementing a method, whereas other studies consider the teacher as *designing* new learning situations. Another category addresses how teachers *lead colleagues* in achieving changes in their schools through their work. Additionally, three dichotomous categories emerged from this dimension: (1) whether contextual factors are discussed in the analysis of teacher competence, which is illustrated as the dichotomy *isolation/context*; (2) whether collaboration skills are seen as part of competence, which is illustrated as the *individual/teamwork* dichotomy; and (3) whether it is argued that different (groups of) teachers have varying digital competences (*specialist/generalist*).
D3: The *attitudes, knowledge and skills* dimension is simply operationalised as whether an article explicitly mentions teachers' *attitudes, knowledge* and *skills* as elements of their digital competence.
D4: The *sources of competence* dimension illustrates the basis of teachers' competence. In the present work, we have identified categories for articles explicitly mentioning *policy*, *theory* or *ethics* as sources of competence. Furthermore, we identified categories based on whether *evidence from research* or *from experience* as sources of competence is explicitly mentioned along with *norms.* Finally, the dichotomous category *global/local* is used to characterise whether competence is reported to be based on global or local sources.
D5: *Relationship to disciplinary content* is the dimension that illustrates the ways in which teachers' digital competence is related to disciplinary subjects or has more generic forms. Competence may be related to a specific subject or mentioned as relevant to subjects without specification. We identified four categories in this dimension: (1) *within subject (specific),* where competence is related to a specific subject; (2) *within subject (unspecific),* where competence is related to a subject that is not specified in the article; (3) *without subject,* where no subject is mentioned; and (4) *subject as a factor*, where the subject is mentioned as a factor in relation to competence.
D6: The *assessment* dimension describes the ways in which teachers' competence is identified and assessed. Some studies have emphasised that teachers' digital competence can be assessed through *self-report surveys and interviews*, while others have argued that it can only be fully assessed by *observing a teacher in a classroom setting.* We have also identified a category based on two aspects: (1) whether teachers' digital competence is represented by *simple models* or (2) whether the competence is too complex to be measured by simple models and must be looked at inductively based on the teacher in question (one teacher at a time). Finally, this dimension is categorised by whether the article has a normative perspective on teachers' competence, thus describing what teachers ‘ought to know’ or whether it is more descriptive of the competence possessed by some teachers and what they ‘really do’. The final coding scheme and its operationalisations are included in Appendix A.

## 5 Results

**Fig. 1:**
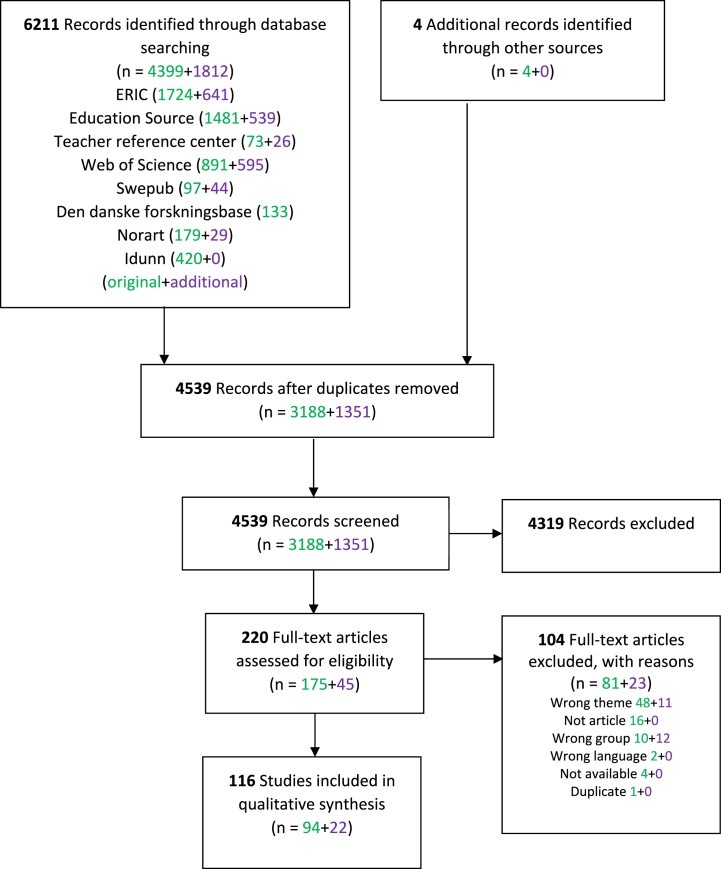
PRISMA diagram. Original search in green; additional search in purple.

We reviewed a total of 4539 titles and abstracts, among which we read 220 full-text articles and ultimately included 116 articles. The PRISMA diagram in Fig. 1 presents more details, including the numbers for the original and additional searches. For more information about PRISMA diagrams, see Moher et al. [22].

Among the 116 articles, 94 and 22 articles published before and during the COVID-19 period, respectively, were included (see Table 1, Table 2, Table 3, Table 4, Table 5, Table 6). The unit of analysis is the teachers' (or student teachers') competence. The articles were read and analysed using the categories identified within the six dimensions described by Smestad and Gillespie [7]. Some categories within each dimension are not mutually exclusive. Furthermore, for each dimension, some articles were identified as explicitly mentioning two or more categories as part of the teachers' (student teachers’) competence. In Table 1, Table 2, Table 3, Table 4, Table 5, Table 6, we did not include all the categories used in the analyses. Instead, we selected only those categories whose frequency merits further discussion; that is, a substantial proportion of the articles (>15%) have addressed them.

**Table 1:** Proportion of articles where the dimension 1 and its categories are mentioned.

Dimensions	Pre (94 pub)	During (22 pub)	Tot (116 pub)
**D1 Beneficiary**			
Individual	28%	18%	26%
Group	40%	45%	41%
Other (social/context)	9%	14%	11%

Note: As the categories are not mutually exclusive, some articles could be identified with two or more categories within the same dimension.

**Table 2:** Proportion of articles where the dimension 2 and its categories are mentioned.

Dimensions	Pre (94 pub)	During (22 pub)	Tot (116 pub)
**D2 Teachers' role**			
Functionary	51%	45%	50%
Designing	40%	23%	37%
Context	17%	14%	16%
Individual	17%	14%	16%

Note: As the categories are not mutually exclusive, some articles could be identified with two or more categories within the same dimension.

**Table 3:** Proportion of articles where the dimension 3 and its categories are mentioned.

Dimensions	Pre (94 pub)	During (22 pub)	Tot (116 pub)
**D3 Attitudes/knowledge/skills**			
Attitudes	47%	55%	48%
Knowledge	66%	64%	65%
Skills	70%	64%	69%

Note: As the categories are not mutually exclusive, some articles could be identified with two or more categories within the same dimension.

**Table 4:** Proportion of articles where the dimension 4 and its categories are mentioned.

Dimensions	Pre (94 pub)	During (22 pub)	Tot (116 pub)
**D4 Sources of competence**			
Policy	6%	27%	10%
Theory	35%	23%	32%
Evidence (experience)	44%	41%	43%
Local	21%	23%	22%
Global	9%	18%	10%

Note: As the categories are not mutually exclusive, some articles could be identified with two or more categories within the same dimension.

**Table 5:** Proportion of articles where the dimension 5 and its categories are mentioned.

Dimensions	Pre (94 pub)	During (22 pub)	Tot (116 pub)
**D5 Relation to disciplinary content**			
Within subject specific	24%	5%	21%
Within subject unspecific	19%	9%	17%
Without subjects	36%	73%	43%

Note: As the categories are not mutually exclusive, some articles could be identified with two or more categories within the same dimension.

**Table 6:** Proportion of articles where the dimension 6 and its categories are mentioned.

Dimensions	Pre (94 pub)	During (22 pub)	Tot (116 pub)
**D6 Assessment**			
Self-reported	56%	77%	60%
Observed in a classroom setting	19%	5%	16%
Models	47%	23%	42%
‘Ought to’	21%	0%	18%

Note: As the categories are not mutually exclusive, some articles could be identified with two or more categories within the same dimension.

Below, we present more detailed results for each dimension with selected references and then discuss these results.

### 5.1 The beneficiary dimension

*D1:* The *beneficiary* dimension refers to explicit mentions or examples of who benefits from teachers' possession of digital competence. Almost 70% of the 116 articles (the full material) explicitly mention who benefits from teachers' digital competence. Further, 41% explicitly address how a group of pupils benefit from such competence, while 26% explicitly mention individual pupils and 11% mention others (society or other contexts).

When comparing articles published before and during the COVID-19 pandemic (see Table 1), there is a higher percentage of articles explicitly mentioning benefits for individuals before COVID-19 (28%) compared with during the pandemic (18%). There are rather small differences (40% vs. 45%) when it comes to articles explicitly mentioning the benefits obtained by a group.

First, starting with all articles (before and during COVID-19), most that explicitly address who benefits from teachers' digital competence mention a group of pupils. In that sense, teachers' digital competence is seen as generally addressing the class without any specific target group [see, e.g. Refs. [[23], [24], [25]]]. This might relate to the idea that digital competence is generic and ubiquitous (see dimension 5). Studies indicating individual pupils as beneficiaries typically refer to ‘digital competence’ as the ability to apply specialised technology to arrange for adapted teaching [see, e.g. Refs. [[26], [27], [28]]]. However, it must be noted that studies on special needs teachers were excluded from the search.

Second, the relatively large share of articles (32%) not explicitly mentioning any beneficiary suggests that having digital competence is expected in general, without stating any particular purpose. This might be grounded in a policy reason for teachers to develop their digital competence, namely being prepared for education for future competences [see, e.g. Refs. [23,29,30]].

The increased share of studies focusing on groups and reduced share of those focusing on individuals illustrate an interesting development. This may be because many teachers experienced internet-based or online learning during the pandemic, thereby implementing digital solutions for whole groups of pupils. This was a relatively new situation for many teachers [see, e.g. Refs. [[31], [32], [33]]]. Consequently, many of them may have been busy learning and managing relevant technologies and have not had the extra capacity to work with their students’ individual needs.

### 5.2 The teachers’ role dimension

*D2:* This dimension concerns which *teacher role* the digital competence allows for. The teachers' role is most often mentioned as functionary (50% of the full material), although designing teaching is also mentioned in many articles (37%; see Table 2). Only a small proportion of the articles mentions how digital competence can allow for leadership (4%). In addition, more articles see teachers working as individuals (16%) than in teams (9%), and 16% of the articles discuss teachers' varying contexts. However, approximately 30% of the reviewed articles do not clearly describe the intended teacher role.

Moreover, there is a reduced percentage of articles explicitly mentioning designing (from 40% to 23%) from the period before to during the COVID-19 pandemic, whereas there are rather small differences in those mentioning functionary (from 51% to 45%), individual and context (both from 17% to 14%). Additionally, some articles [see, e.g. Refs. [31,34,35]] from the COVID-19 period mention emergency remote teaching (ERT) as a concept to describe how teaching with digital tools was conducted during the pandemic.^1^

The fact that almost every third article does not discuss different teacher roles is of interest. It is rather unlikely that the roles of functionary, designer or leader demand the same competences. Therefore, discussions of teachers' digital competence would benefit from explicit references to these roles. Meanwhile, there is a large proportion of articles addressing teachers' role as functionaries. Teachers' use of predesigned technologies dominates these articles [[36], [37], [38]]. In this sense, digital competence is understood as employing a standard educational design in terms of using commercially developed teaching materials. Consequently, a relatively large share of articles addressing competence in designing teaching should be investigated further. This aspect seems to imply that a key part of teachers' digital competence lies in designing the kind of learning environment needed to improve their teaching and provide benefits for their pupils; it also relates to a strong understanding of teachers’ autonomy [see, e.g. Refs. [25,39]]. This might also indicate that digital technology plays an important role in forging innovative approaches to teaching and learning. However, the decrease in the share of articles addressing design during the COVID-19 pandemic is particularly disheartening. This might be explained by the fact that ERT largely entails coping with technology as a distribution channel rather than developing a new and innovative technology-based pedagogy.

The other subelements of this dimension, such as context, individual or teamwork, are mentioned in less than 20% of the articles [see, e.g. Refs. [38,40,41]]. This might reflect the general impression that teachers’ professional digital competence is implicitly judged as mainly technical and not related at all to context or social relations.

### 5.3 Attitudes, knowledge and skills dimension

*D3:* This dimension is related to the three categories of competence (attitude, knowledge and skills) included in teachers' professional digital competence. Overall, 69%, 65% and 47% of the articles mention ‘skills’, ‘knowledge’ and ‘attitude’, respectively (Table 3). These percentages illustrate how some articles emphasise two or all three elements of competence. The percentage of articles explicitly mentioning ‘attitude’ shows an increase from 47% pre-COVID-19 to 55% during COVID-19; accordingly, the percentage of those mentioning ‘skills’ shows a decrease of 6% points. There are minor differences in the percentage mentioning ‘knowledge’.

The high share of articles addressing skills and knowledge when studying teachers' digital competence is in line with the prevalent understanding of digital competence as comprising practical skills and concrete knowledge of technologies; it is often described in models and operationalised into concrete ‘know-how’, as is firmly documented in studies devoted to TPACK [2] and DigCompEdu [3]. In the reviewed articles, we have identified some differences in the articles regarding attitudes and skills published before and during the pandemic. These changes are not major and may be coincidental. During the pandemic, many studies on practical experiences with ERT were published [see, e.g. Refs. [32,34,35]], highlighting cases of digital citizenship and teachers' possible reluctance to use digital technologies. Teachers' attitudes towards the pedagogical use of technology may change when they see utility through their ERT, which might be essential in changing attitudes towards technology use. This is in line with the study of McDonagh et al. [16], who emphasised the attitudinal dimension as imperative in defining teachers' digital competence.

### 5.4 The source of the competence dimension

*D4:* Some of the articles describe ‘sources of competence’, that is, the basis for teachers' competence. In the full material, 43% mention evidence experience, while 32% mention theory (Table 4). Further, 22% and 10% of the articles describe the source of competence as local or global, respectively. There is a decrease in the number of articles that view theory as a source of competence (from 35% to 23%) and an increase in the number of articles discussing the concept of global competence (from 9% to 18%). Otherwise, there are only small differences.

Overall, the reviewed articles indicate that evidence by experience represents the largest source of teachers' digital competence. This is in line with a long history of teacher research showing that teachers' own experiences and evidence from colleagues comprise an important basis for competence [42]. Norwegian teachers also emphasise professional development activities related to their own experiences [ [43], p. 17]. The fair share of theory as a source of competence might relate to the fact that many articles lend themselves to the TPACK model as a framework for investigating teachers’ digital competence [see, e.g. Refs. [[44], [45], [46]]]. We believe that the decrease in the share of theory as a source of competence in articles published during the pandemic calls for further investigation. One explanation might be that the ERT approach demands quick solutions and less theoretically founded approaches in the development of relevant digital competences. One example is the use of YouTube for instructions on how to use new technology, given the lack of immediate access to colleagues or experts when searching for solutions.

### 5.5 The relationship to the disciplinary content dimension

*D5:* This dimension deals with how teachers' digital competence is related to disciplinary content in a specific subject. In the full material (Table 5), 43% of the articles do not mention a subject; in comparison, 21% discuss teachers' digital competence within one or more specific subjects. About 17% of the articles mention the importance of the subject while being unspecific about what subject they have in mind. Overall, there seems to be a higher percentage of articles published during the pandemic that do not mention a subject compared to those written prior. In line with this finding, the number of articles mentioning subjects, whether specific or not, shows a decrease.

As mentioned, a substantial share of articles does not address a specific subject in their research [see, e.g. Refs. [[47], [48], [49]]]. On the one hand, this is surprising, not least because of the prevalence of the use of the TPACK model, in which content is an area. On the other hand, there are articles using the TPACK model where content is not specified [see, e.g. Refs. [44,50,51]]. However, several articles discuss subject-specific areas [see, e.g. Refs. [24,52,53]]. These differences indicate differences in approaches in terms of defining teachers’ digital competence related to subjects: 1) generic definitions expanding existing models [see, e.g. Refs. [26,54]] and 2) specific definitions relevant to particular subjects [see, e.g. Refs. [38,52,55]]. In the context of ERT, teachers rely on digital tools in all subjects simultaneously. This may help explain why there is less interest in particular subjects in the articles published during the COVID-19 pandemic.

### 5.6 The assessment dimension

*D6:* This dimension concerns how teachers' digital competence can be identified and determined. In the full material (Table 6), 60% of the articles are based on teachers' self-reported data, while 16% use observation as their data collection method. The amount of self-reported data shows an increase from 56% before COVID-19 to 77% during the pandemic. Observation, however, is less used during the pandemic, with its percentage shrinking from 19% to 5%.

As mentioned by Smestad and Gillespie [7], many studies on teachers’ digital competence have utilised models. In the analysed material, 42% of the articles use models, but this percentage shows a decrease from 47% before COVID-19 to 23% after the pandemic. About 18% of the articles use a normative approach, discussing what the teachers should do in the classroom. In comparison, in the materials published during COVID-19, we did not find any of these explicit normative examples.

Moreover, a large share of the articles based their analysis on self-reported data [see, e.g. Refs. [49,50,56]]. This is a well-known weakness in measuring and investigating digital competence because self-reported data—particularly within a competence characterised by practical skills—often do not represent actual competence [57]. The extended use of models (e.g., TPACK) and associated questionnaires for investigating teachers' digital competence is probably the reason behind the high number of articles based on self-reported data, which is demonstrated by the large number of articles based on models in the full material [see, e.g. Refs. [26,32,58]]. One initiative to ameliorate this kind of weakness in validity was introduced by Harris et al. [59], who developed an observational tool to capture actual rather than self-reported competence. The fact that there are no significant differences in the categories of ‘ought to’ and ‘do’ indicates that both normative and descriptive approaches are included in the material [see, e.g. Refs. [31,51,60]].

The increase in self-reported data and decrease in observation in articles published during the pandemic compared with those published before can be explained by practical circumstances. Self-reported data are more easily generated and available than classroom observations. Although classroom observations are, theoretically, only a mouse click away in the case of digital teaching, practical and privacy concerns pose challenges to employing this method. At the same time, qualitative studies take longer to perform than quantitative studies; hence, qualitative, observation-based studies conducted during the COVID-19 pandemic may have not yet been published.

Finally, the decrease in articles explaining digital competence using models may be explained by changes in what is deemed relevant during home schooling and ERT. In particular, most models are designed to describe the situation in physical classrooms, not in online teaching situations; thus, they might not be relevant to the pandemic situation.

## 6 Discussion

The present paper has addressed (student) teachers' digital competence, as mentioned explicitly in 116 articles (94 published pre-COVID-19) together with the elements that characterise descriptions of this competence within six dimensions. The research builds on the dimensions of the three transdisciplinary competences identified by Smestad and Gillespie [7]. Furthermore, the current article contributes by designing categories that describe teachers' digital competence in greater detail. In this discussion, we highlight three larger issues: the unclarity of many articles concerning the beneficiaries of digital competence, the teachers' role and the relation to subjects; the predominance of the role of functionary rather than designer; and researchers’ strong reliance on self-reported data. We also discuss developments that occurred because of COVID-19.

It is surprising that many of the articles do not discuss what kind of teacher role (functionary, designer or leader) is assumed, do not mention who the beneficiaries of digital competence are and do not go over which subject(s) the competence is relevant for. These findings may signify that either researchers believe there is no need to mention these aspects (because they are obvious) or view teachers’ digital competence as something generic and the same, regardless of teacher role, beneficiary or subject.

When it comes to beneficiaries (D1), it can be argued that this has been discussed so thoroughly (e.g., in policy documents) that one can take for granted that all pupils in general are beneficiaries, especially with schools aiming to develop pupils’ twenty-first-century skills. When articles mention beneficiaries, groups of pupils are more often addressed than individual ones. Therefore, we can conclude that digital competence is viewed as generic and that this does not demand different competences for specific groups of pupils.

The same cannot be said about the teacher's role (D2). There is no consensus that teachers should act as functionaries adopting technologies that come their way, even though this is the teacher role that is most often found in the material. Therefore, considering that some articles view teachers as designers of teaching whose professional work is based on teacher autonomy [61], it is worrying that many articles do not discuss the teacher role at all, even though it is key to understanding teacher competence.

We also question the large proportion of articles that do not mention specific subjects (D4) when discussing teachers' digital competence. This is particularly interesting when considering the large amount of research that employs the TPACK model, in which content is one of three areas. Even articles related to TPACK often leave the subject unmentioned. Thus, teachers’ digital competence has generally been discussed as generic with respect to subjects.

Thus, one major question is whether it makes sense to discuss teachers' digital competence without connecting it to a particular way of handling technologies or to certain beneficiaries and subjects. Are there reasons to believe that teachers with different roles (functionary, designer or leader) need the same competences? Furthermore, do teachers of different subjects need the same digital competence? We argue that teachers’ digital competence should be further investigated while considering these characteristics.

Self-reported data dominate the research on teachers' digital competence, and this is a known weakness in reporting actual competence [62]. This may be partly attributed to the fact that the collection of large amounts of data is more feasible with surveys and that established instruments exist for studying teachers' competence via surveys. However, the surveys need to be short, which may lead to the exclusion of many important factors, such as the teachers' role in connection to technology, the beneficiaries or the importance of the subject. This contributes to the view of digital competence as a generic concept. Often, the specific teaching context is completely absent [63]. Therefore, we recommend that studies include other data sources when discussing teachers' digital competence. Some examples of relevant alternative options include (classroom) observation, document analysis of teaching plans or pupils’ texts and an investigation of learning analytics data.

A dominant source of competence in the material is experience, which is also known in other areas of research on teacher competence [43]. On the other hand, theory is considered a source of teachers' competence, and teachers are supposed to know about TPACK. One can ask why this has become such a dominant theoretical source of teachers’ digital competence when we know that there are other large-scale international (DeSeCo) and European (DigCompEdu) frameworks describing what constitutes digital competence. Is it perceived to be simpler than other models or more useful for teachers, or is this because of its research-based foundation, which is contrary to other models that are more political?

Moreover, the articles published during the COVID-19 period indicate less of a focus on certain groups of pupils, and this might be explained by the lack of capacity to handle more than the whole class when promptly turning to ERT. The ERT situation can also explain why there was a small decrease in research addressing the role of teacher as designer. During the pandemic, there have been more than enough challenges in addressing the ERT situation itself, thus giving teachers less time and capacity to design learning materials. However, the increased use of technology during this period also seems to have changed researchers' interest in teachers' attitudes towards technology, as illustrated in the small increase in articles mentioning attitude as a category. Being forced to use technology may have certainly affected teachers’ attitudes; however, for some, this may be in the direction of motivation [see, e.g. Ref. [31]] and, for others, demotivation [see, e.g. Ref. [64]].

The situation during the COVID-19 pandemic also seems to have reduced the tendency to apply theoretical models and include subject-specific content. This may be because of the ERT situation, which is characterised by the immediate implementation of ready-made solutions, therefore resting on solutions at hand, such as collegial help and the use of generic technology for communication. Finally, the articles published during the COVID-19 period show a decrease in observational data as evidence for competence. This can be explained by restrictions on physical gatherings, making survey data more feasible. Would other conclusions have been drawn if observational data were collected, and will such data collected post-COVID-19 contrast the observational data assessed before the pandemic? It will be interesting to investigate the long-term effects of teachers’ COVID-19-related experiences on their digital competence. In fact, in some articles, we already see signs of a wider concept of digital competence, as teachers are preoccupied with (digital) classroom management, keeping in touch with vulnerable children and other similar aspects during ERT [65].

## 7 Concluding remarks

After discussing the limitations of our study (7.1), we provide conclusions (7.2) and some reflections on lines of research that should be investigated considering our results (7.3).

### 7.1 Limitations

The selection of scope and methodology in the present paper resulted from a series of compromises. A more extended time frame could have yielded more robust findings. Additionally, the exclusion of languages other than English and the three Nordic languages—Norwegian, Danish and Swedish—may have resulted in the omission of significant research in other languages. Similarly, the exclusion of articles that did not explicitly signal their discussion of teachers’ competence in their title or abstract may have resulted in overlooking research addressing competence indirectly.

On the other hand, the analysis could have been more thorough in several ways. For example, if more researchers had read each article, the reliability of the findings could have increased. Additionally, the six dimensions proposed by Smestad and Gillespie [7] provided a direction for the analysis, and although the subdimensions were developed further based on the conceptions of digital competence found in the reviewed articles, starting from a different point might have revealed different tendencies.

Even with these limitations, we argue that such a review is worthwhile in pointing out tendencies and giving food for thought.

### 7.2 Conclusion

The results of the current study indicate that the included research on teachers' digital competence tends to have the following characteristics: it lacks clarity regarding the beneficiaries of digital competence; it positions the teacher more as a functionary than as a designer; it is concerned with teachers' attitudes, knowledge and skills; and it views competence as being based on experience and theory, is often not connected to subjects and is typically based on self-reported data. These characteristics of numerous studies on teachers’ digital competence open opportunities for further discussion.

Having a comprehensive understanding of how researchers view teachers' digital competence can be valuable in various ways. For instance, it can facilitate discussions within the field about potential gaps in current research on this type of competence. Future research should incorporate more classroom observations, emphasise teachers' roles as designers and focus on the beneficiaries of teachers' competence. It would also be crucial to investigate whether teachers’ digital competence is portrayed differently when data are collected through classroom observations instead of questionnaires.

Moreover, this overview can aid in comparing the field of teachers' digital competence with that of other competence areas. We have observed that there is a tendency to overlook the beneficiaries of teachers' digital competence, such as which pupils in which contexts benefit from specific aspects of this competence. This contrasts sharply with the research on teachers' diversity competence, which frequently addresses this issue [see Ref. [7]]. We contend that discussions in the field could gain additional nuance if pupils are viewed less as a homogenous group that will all benefit equally from teachers’ digital competence.

The results of the additional search we conducted suggest that the COVID-19 pandemic changed the picture to a certain extent. The new context of having to teach an entire class digitally over an extended period of time (ERT) may have increased the focus on the whole group of pupils and on employing ready-made educational materials instead of looking into the role of teachers as designers. It may also have increased researchers' interest in local experience as a source of competence and in digital competence across subjects, as opposed to that within particular subjects. In addition, the ‘lockdowns’ may have made classroom observations more difficult for researchers, leading to an even greater reliance on self-reported data.

### 7.3 Further research

Having an awareness of the potential impact of COVID-19 on teachers' digital competence and the associated literature could spark new ideas about which aspects of this competence require further investigation in the near future. The COVID-19 pandemic amplified tendencies that were already evident in research on teachers' digital competence. As a result, there is a stronger need for research based on classroom observations, which may generate new knowledge on what teachers' digital competence constitutes. Furthermore, there is a need for greater emphasis on teachers' roles as designers. For example, what makes teachers designers? The present review study has also revealed a need to focus on the beneficiaries of teachers' competence. Can research focusing on different groups of pupils' outcomes of teachers' digital competence alter the understanding of what this competence constitutes? Finally, the findings of the current study suggest that there is a need for attentiveness to whether teachers’ digital competence is a generic competence or specific to subjects.

In our review, the ERT caused by COVID-19 stood out. It would be interesting to further investigate what the long-term consequences of ERT will be.

## Author contribution statement

All authors listed have significantly contributed to the development and the writing of this article.

## **Acknowledgement**

This work was supported by the Norwegian Research Council (Grant No. 286993) as they funded our project Teacher Qualification for the 21st century - teachers professional competence (TEQ21). We also would like to thank the TEQ21 research group in general, and in special Hanne Christensen and Astrid Gillespie for their contribution to the review.

## Data availability statement

Data will be made available on request.

## Additional information

Supplementary content related to this article has been published online at [URL].

## Declaration of competing interest

The authors declare that they have no known competing financial interests or personal relationships that could have appeared to influence the work reported in this paper

## Footnotes

1 Although mentioned only thrice in the material on teachers' digital competence, ‘emergency remote teaching’ has become a frequently used phrase, with more than 10,000 hits on Google Scholar as of May 2022.
