# Supplementary material for: Examining dimensions of teachers’ digital competence: A systematic review pre- and during COVID-19
Source: Heliyon. 2023 May 26;9(6):e16677. doi: 10.1016/j.heliyon.2023.e16677 (PMC10245064; doi:10.1016/j.heliyon.2023.e16677)
Supplement: Multimedia component 1 [file mmc1.docx]

Appendix A shows the dimensions and categories used to analyse the teacher’s competence in the articles. The dimensions were developed by Smestad & Gillespie (2020), and the categories were developed by the authors of this article.

| **Dimensions and categories** | **Descriptions** | |
| --- | --- | --- |
| **D1 Beneficiary dimension** | **D1 concerns who is to benefit from the teacher having this competence.** | |
| Individual | It is explicitly said that there are particular pupils who are to gain benefit from the teachers’ competence. (Example: the learning of a blind student is in focus.) | |
| Group | It is explicitly said that groups of pupils gain benefit from the teachers’ competence. | |
| Social | It is explicitly said that society gains benefit from the teachers’ competence. | |
| Context | Teachers’ competence is seen to include the ability to connect to pupils’ context (home and family, culture, religion…) | |
|  |  | |
| **D2 Teachers’ role** | **D2 concerns which teacher role teachers digital competence (tDIGc) allows for.** | |
| Functionary | The teacher uses technology which others have created and decided how to use. | |
| Designing | The teacher designs (alone or with others) new learning situations. | |
| Leadership | The teacher leads colleagues in change work in school. | |
| Isolation | No contextual factors are mentioned in the analysis of teachers’ competence. (Typically, the data are collected via individual questionnaires) | |
| Context | The teachers’ competence is seen as contextual. | |
| Individual | Collaboration skills (the teachers’ competence in working with others) are not seen as part of tDIGc. | |
| Teamwork | Collaboration skills are seen as part of tDIGc. | |
| Specialisation | It is argued that different groups of teachers will have different “versions” of tDIGc. | |
| Generalist | tDIGc is considered a general “package of” competence that all teachers need. | |
|  |  | |
|  |  | |
| **D3 Attitude, knowledge and skills** | **D3 considers the “kinds” of competence that are included in tDIGc** | |
| Attitudes | Attitudes are explicitly mentioned as an element of tDIGc. | |
| Knowledge | Knowledge (in the sense of facts and concepts) is explicitly mentioned as an element of tDIGc. | |
| Skills | Skills are explicitly mentioned as an element of tDIGc. | |
|  |  | |
|  |  | |
| **D4 Sources of competence** | **D4 is what a teachers’ competence is based upon** | |
| Policy | Policies are explicitly mentioned as sources of competence | |
| Theory | Theory is explicitly mentioned as a source of competence | |
| Ethics | Ethics is explicitly mentioned as a source of competence | |
| Evidence (research) | Research evidence is explicitly mentioned as a source of competence | |
| Evidence (experience) | Experience is explicitly mentioned as a source of competence | |
| Norms | Norms are explicitly mentioned as sources of competence | |
| Global | Teachers’ competence is based on global sources | |
| Local | Teachers’ competence is based on local sources | |
|  | |  |
| **Dim. 5 Content** | **Relationship to disciplinary content** | |
| Within subject spesific | Teachers’ digital competence to be teacher in a specific subject. | |
| Within subject unspecific | Mentioned that the competence is relevant to subjects, but this is not specified. | |
| Without subjects | No specific subject is mentioned | |
| Subject as a factor | The teacher’s subject is used as an argument for use/non-use | |
|  |  | |
| **D6 Assessment** |  | |
| Self-reported | Data is collected as surveys, interviews and other kinds of self-reported data | |
| Observing in classroom setting | The data on the competence is retrieved through classroom observation | |
| Testing | The data on competence is retrieved through tests. | |
| Models | Models such as TPACK and other kind of frameworks forms the basis of how tDIGc is measured/evaluated | |
| One teacher at a time | Case studies employed to understand tDIGc without referring to simple models such as TPACK (inductive) | |
| Ought to | The article is normative on teachers´ competence | |
| Do | The article is descriptive on teachers´ competence | |

*Note: The categories are not mutually exclusive.*
